# A clinical study of peroral endoscopic myotomy reveals that impaired lower esophageal sphincter relaxation in achalasia is not only defined by high-resolution manometry

**DOI:** 10.1371/journal.pone.0195423

**Published:** 2018-04-02

**Authors:** Hiroki Sato, Kazuya Takahashi, Ken-ichi Mizuno, Satoru Hashimoto, Junji Yokoyama, Shuji Terai

**Affiliations:** 1 Division of Gastroenterology, Niigata University Medical and Dental Hospital, Niigata, Japan; 2 Department of Gastroenterology, Saiseikai Niigata Daini Hospital, Niigata, Japan; Hokkaido University, JAPAN

## Abstract

**Background and aim:**

Achalasia is an esophageal motility disorder characterized by impaired lower esophageal sphincter (LES) relaxation. On high-resolution manometry (HRM), impaired LES relaxation is defined by elevated integrated relaxation pressure (IRP). However, a new category of achalasia within the normal IRP range has been suggested.

**Methods:**

HRM was performed using a Starlet device and an IRP threshold of 26 mmHg. Peroral endoscopic myotomy (POEM) was performed for cases of achalasia diagnosed using established methods. During POEM, the histology of the LES was assessed. Follow-up was performed 2 months post-operatively.

**Results:**

Forty-one patients with achalasia (18 women, mean age 53 ± 18.6 years) were included. Among them, 27 were placed in the IRP > 26 mmHg subgroup (impaired LES relaxation on HRM) and 14 in the IRP ≤ 26 mmHg subgroup (normal LES relaxation on HRM). In the IRP ≤ 26 mmHg subgroup, patients were older, had longer symptom duration, and had more esophageal dilation. The IRP ≤ 26 mmHg subgroup had the same symptom severity as the higher IRP subgroup and POEM significantly improved symptoms and IRP, although four patients still had severe LES fibrosis.

**Conclusions:**

The clinical presentation of achalasia has a gap between a HRM-defined impaired LES relaxation, with aging or disease progression considered reasons for a lowered LES pressure. POEM can be a feasible treatment option, even for cases of achalasia with a normal IRP. However, patients with severe LES fibrosis need more attention for the therapeutic indication.

## Introduction

Achalasia is a major esophageal motility disorder characterized by impaired lower esophageal sphincter (LES) relaxation and a lack of normal peristalsis in the esophageal body.[1] Patients with achalasia experience chronic esophageal symptoms such as dysphagia, regurgitation, and chest pain.[2]

**Fig 1:**
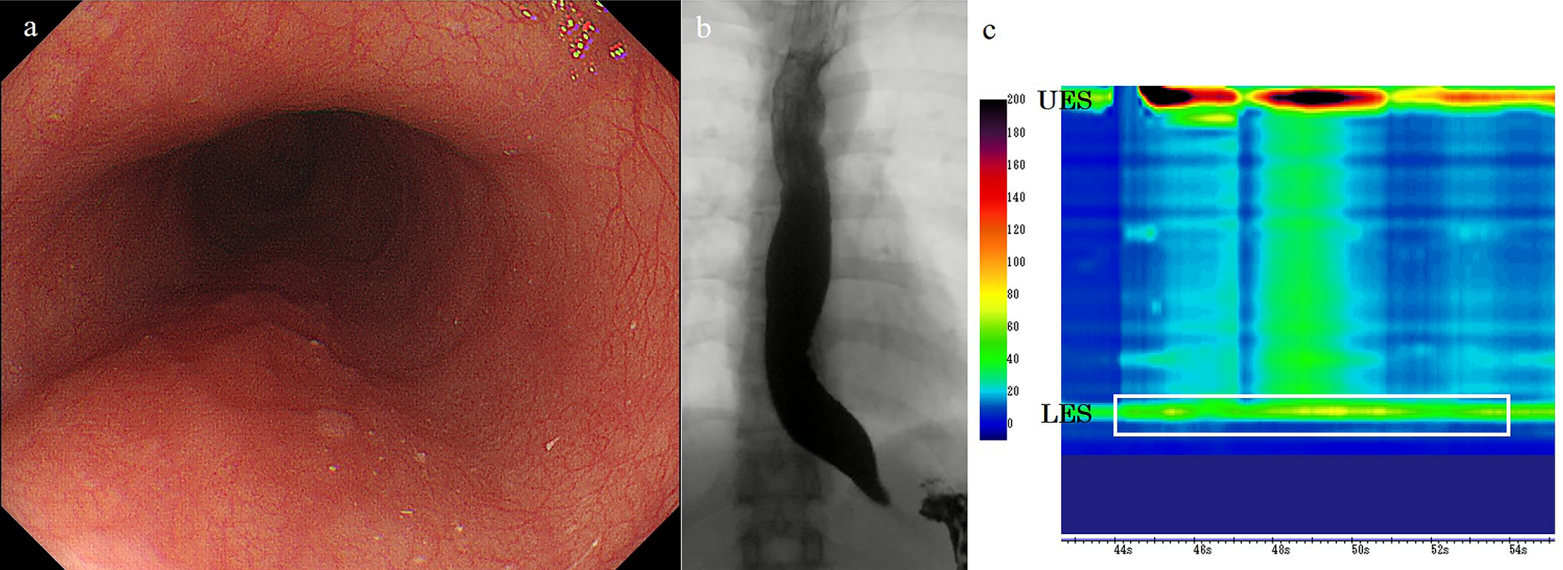
(a) On endoscopy, the esophagus looks normal without dilation. However, increased resistance was observed through the esophago-gastric junction in a patient with achalasia. (b) Esophagography showing a bird-beak appearance with remnant barium in a non-dilated esophagus. (c) High-resolution manometry showing pan-esophageal pressurization (type-II achalasia) with elevated integrated relaxation pressure (IRP) (43.3 mmHg; the IRP measurement was taken after deglutitive upper sphincter relaxation, based on the 4-s window in which the e-sleeve value is lowest, noting that the 4 s did not have to be continuous, but could be distributed within a 10 s time window (white closing box).

Manometry plays an important role in the differential diagnosis of esophageal motility disorders. Based on the development of high-resolution manometry (HRM),[3] the Chicago classification criteria were proposed and are used as the gold standard for the diagnosis of esophageal motility disorders.[4, 5] Integrated relaxation pressure (IRP) is the most important parameter assessed via HRM for evaluating LES relaxation, which is measured after deglutitive upper sphincter relaxation from the anticipation of esophago-gastric junction (EGJ) relaxation until peristaltic wave arrival. An IRP > 15 mmHg is defined as "impaired LES relaxation" and considered as a necessary condition for the diagnosis of achalasia, although the threshold should only be used with the Medtronic system and differs between different devices (Fig 1).[6]

**Fig 2:**
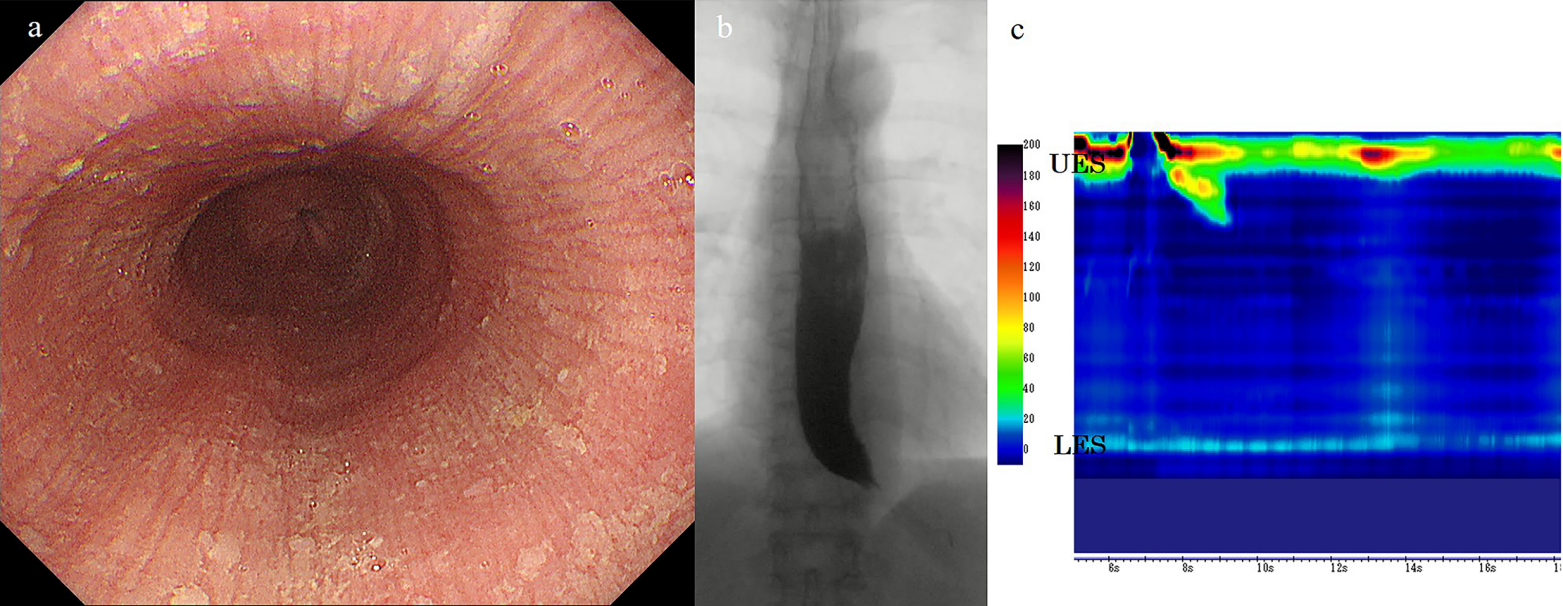
(a) Endoscopy showing a mucosal pinstripe pattern with increased resistance through the esophago-gastric junction.[11] (b) On esophagography, bird-beak appearance and remnant barium in a non-dilated esophagus were observed. (c) High-resolution manometry showing 100% failed peristalsis (type-I achalasia) with normal integrated relaxation pressure (22.9 mmHg).

**Fig 3:**
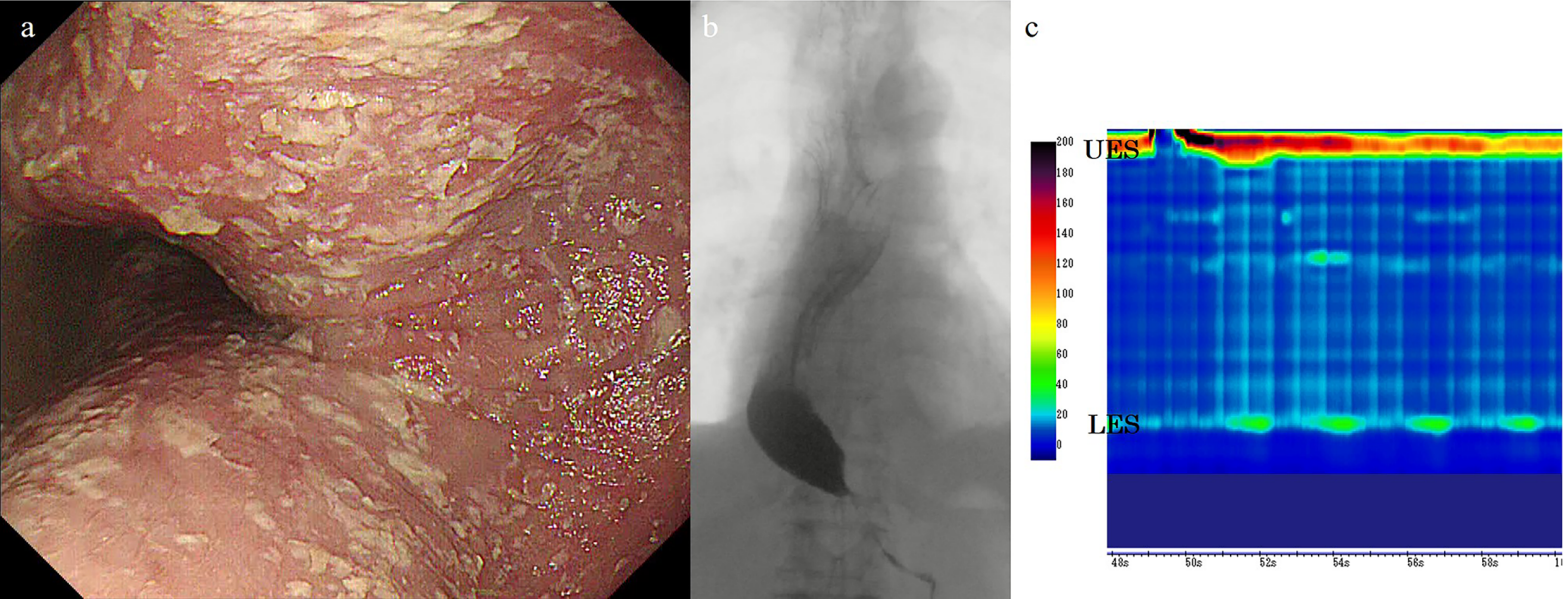
(a) Endoscopy showing a totally dilated esophagus. (b) Esophagography showing sigmoid-like appearance with retention of contrast medium. (c) High-resolution manometry showing 100% failed peristalsis (type-I achalasia) with normal integrated relaxation pressure (18.5 mmHg).

However, Ponds *et al*. recently reported on a series of achalasia patients in whom the LES pressure was normal (IRP ≤ 15 mmHg) and found that impaired EGJ distensibility was the key pathology in this subgroup.[7] In our previous report, 28.1% (9/32) of patients with achalasia had an IRP within the normal range and the HRM findings did not match the typical findings of achalasia seen on endoscopy and esophagography (Figs 2 and 3).[8]

Reports concerning this subgroup of achalasia patients with normal LES relaxation on HRM are limited. With this in mind, this study aimed to clarify the characteristics of this subgroup of achalasia patients by comparing patient characteristics and the therapeutic outcomes of a new minimum invasive endoscopic surgery, peroral endoscopic myotomy (POEM),[9] with those of an original endoscopic technique for the histological assessment of LES.[10]

## Methods

### Statement of ethics

The present study was conducted prospectively with the approval of the Niigata University Review Board. The present study was carried out in accordance with the Declaration of Helsinki. Written informed consent was obtained from all patients prior to their participation in the study.

### Patients

Patients with esophageal symptoms and suspected of having an esophageal motility disorder were referred to our hospital. All the patients were diagnosed using established methods, including upper gastrointestinal endoscopy, esophagography, and HRM using a Starlet device (Starmedical, Tokyo, Japan). On HRM, after catheter intubation, patients were required to perform 10 5-mL water swallows in the supine position. An IRP threshold of 26 mmHg was used to define impaired LES relaxation on HRM.[12] Achalasia was diagnosed as follows: on endoscopy, no anatomical lesions, neoplasia, or increased resistance at the EGJ; on esophagography, bird-beak or sigmoid-like appearance with the retention of contrast medium; and no normal peristalsis on HRM. On endoscopy, the appearance of rosette-like esophageal folds or the presence of the longitudinal superficial wrinkles of the esophageal mucosa (pinstripe pattern) were also noted.[11, 13] Manometry patterns of achalasia were categorized based on HRM as follows: type I (classic achalasia), 100% failed peristalsis; type II, no normal peristalsis, pan-esophageal pressurization during ≥ 20% of swallows; and type III, no normal peristalsis, preserved fragments of distal peristalsis or premature (spastic) contractions during ≥ 20% of swallows according to the Chicago classification criteria. These patients with achalasia were categorized into subgroups: IRP > 26 mmHg (impaired LES relaxation on HRM) and IRP ≤ 26 mmHg (normal LES relaxation on HRM). Patients treated with achalasia balloons, botulinum toxin injections or Heller’s myotomy as well as those who experienced recurrent symptoms, were not included in the study.

Achalasia-related symptoms were assessed using the Eckardt score, which is the sum of the achalasia-related symptom scores for dysphagia, regurgitation, chest pain, and weight loss. The Eckardt score was used to assess the severity of achalasia symptoms and treatment effectiveness: a higher Eckardt score reflects more severe symptoms of achalasia (maximum: 12), while a lower score indicates milder symptoms (minimum: 0).[14, 15] On esophagography, the degree of esophageal dilatation was classified according to the diameter of the esophageal lumen: not dilated (< 3.5 cm) or dilated (> 3.5 cm).[16]

### Peroral endoscopic myotomy

All patients with achalasia defined as above were treated with POEM. POEM was performed based on the technique previously described by Inoue *et al*.[17] During incision of the muscularis propria layer, peroral endoscopic muscle layer biopsy (POEM-b) was performed to evaluate the histology of LES.[18, 19] Fibrosis of the LES was graded as previously reported (grade 1: no to mild fibrosis, grade 2: moderate fibrosis, grade 3: severe fibrosis).[20]

The initial follow-up visit was 2 months post-operatively. Post-operative testing included upper gastrointestinal endoscopy, esophagography, and HRM.

### Statistical analysis

Median ± standard deviations and ratios were used to express continuous variables and categorical variables to describe patient baseline characteristics. These data were compared between subgroups with an IRP > 26 mmHg and IRP ≤ 26 mmHg using Student's t-test and the Mann–Whitney *U*-test for parametric and nonparametric data, respectively. Spearman's correlation coefficient was used to determine the correlation between IRP and parameters with statistical significance in the comparison of patient baseline characteristics.

Eckardt score and IRP before and after POEM were compared and changes in these parameters were assessed with Student's t-test. Significance was assumed at P < 0.05. Statistical analysis was performed using SPSS Version 20 (IBM SPSS Inc, Chicago, III).

## Results

**Fig 4:**
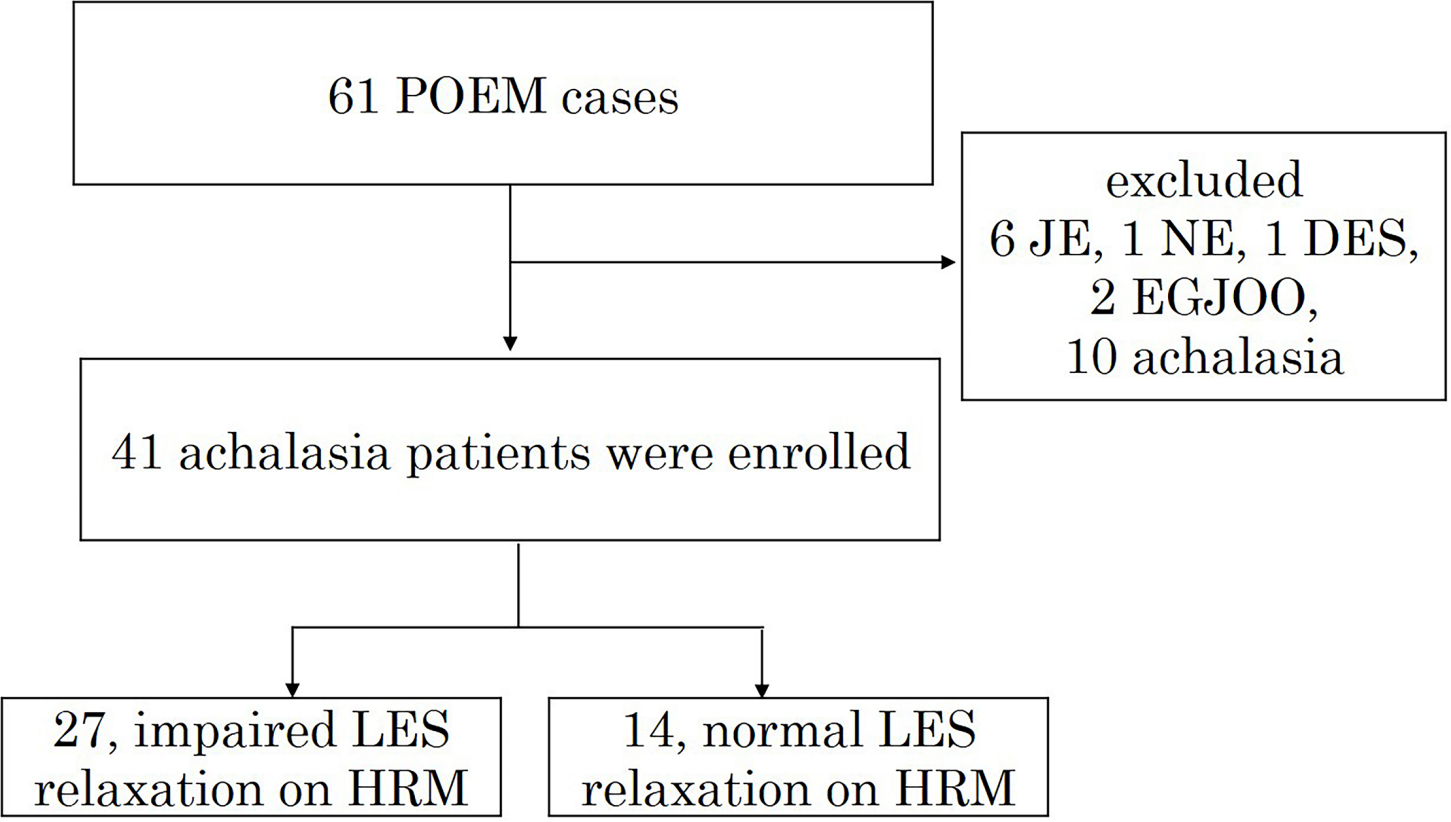
Flowchart of patient enrollment. From 61 cases of peroral endoscopic myotomy, 20 cases were excluded and 41 patients with achalasia were finally enrolled. Thereafter, twenty-seven achalasia patients were categorized into a subgroup with integrated relaxation pressure (IRP) > 26 mmHg (impaired lower esophageal sphincter [LES] relaxation on high-resolution manometry [HRM]), whereas 14 were placed into the IRP ≤ 26 mmHg group (normal LES relaxation on HRM).

Out of the 61 patients who underwent POEM, 41 (18 women, mean age 53 ± 18.6 years) were finally included in the study. Ten patients were excluded because pre-HRM testing could not be accomplished due to failed passage of a catheter through the LES. We ruled out other esophageal motility disorders, such as EGJ outflow obstruction, distal esophageal spasm, and jackhammer esophagus. Based on esophageal body motility, these 41 patients with achalasia were sub-classified into those with type I (classic achalasia, n = 26 [63.4%]), Type II (panesophageal pressurization, n = 11 [26.8%]), and type III (premature contractions, n = 4 [9.8%]). Twenty-seven achalasia patients were categorized into the IRP > 26 mmHg subgroup (impaired LES relaxation on HRM) and 14 into the IRP ≤ 26 mmHg subgroup (normal LES relaxation on HRM) (Fig 4).

### Characteristics of achalasia patients with normal IRP on HRM

Between the two groups with IRP > 26 mmHg and IRP ≤ 26 mmHg, the following data were compared: sex, age, body mass index (BMI), duration of symptoms, Eckardt score, pattern of manometry, and degree of esophageal dilation (Table 1).

**Table 1:** Patient characteristics.

Patient characteristics	Achalasia (n = 41)	Impaired LES relaxation on HRM (n = 27)	Normal LES relaxation on HRM (n = 14)	P-value
Sex ratio (Male:female)	23:18	16:11	7:7	NS (0.63)
Age, years^†^	53 ± 18.6	46 ± 19.3	64.5 ± 11.8	0.01
Body mass index^†^	20.0 ± 3.9	18.9 ± 3.9	20.7 ± 3.3	NS (0.11)
Duration of symptoms, years^†^	5.0 ± 9.3	3.7 ± 6.0	8.4 ± 12.1	0.01
Eckardt score for achalasia^†^	7.0 ± 2.3	7.0 ± 2.1	7.0 ± 2.6	NS (0.35)
Achalasia type (I:II:III), n	26:11:4	15:9:3	11:2:1	NS (0.23)^‡^
Degree of esophageal dilation (not dilated:dilated), n	24:17	20:7	4:10	0.02
Integrated relaxation pressure, mmHg^†^	29.3 ± 14.7	38.6 ± 13.3	21.5 ± 5.0	0.00
Residual LES pressure, mmHg^†^	32.0 ± 14.1	37.9 ± 14.0	19.4 ± 6.9	0.00

^†^Median (standard deviation). LES = lower esophageal sphincter; HRM = high-resolution manometry; NS = not significant.

^‡^P-value was calculated between type I and II+III

In the IRP ≤ 26 mmHg subgroup, patient age and symptom duration were significantly higher and longer (P = 0.01, 0.01, respectively). Moreover, the esophagus was more dilated (P = 0.02). Sex, BMI, Eckardt score, and manometry pattern were not significantly different between the groups.

**Fig 5:**
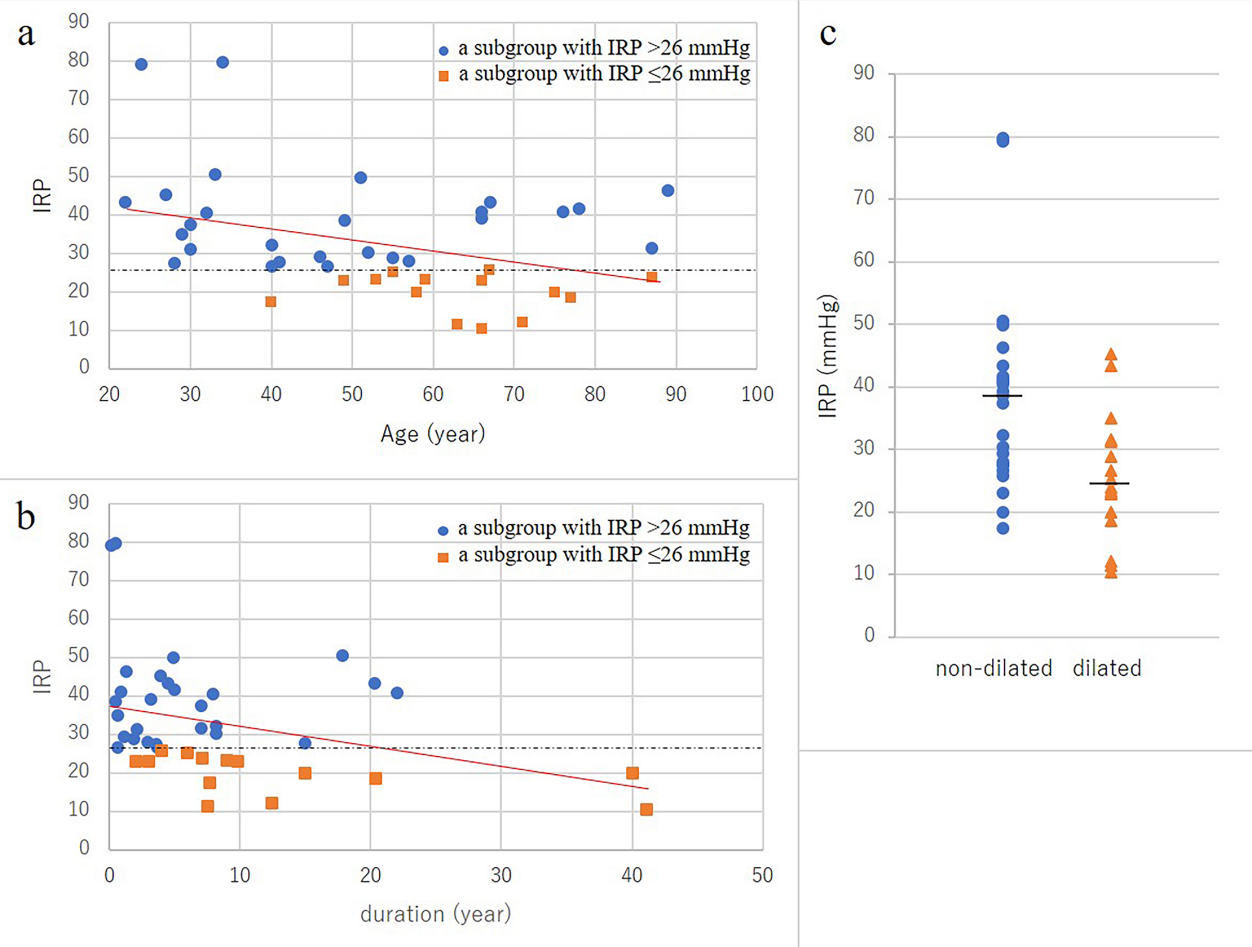
Spearman's correlation coefficient to determine the correlation between integrated relaxation pressure (IRP) and age (Fig 5a) or IRP and symptom duration (Fig 5b), calculated as -0.308 (P = 0.05) and -0.371 (P = 0.02), respectively. The dotted horizontal line means IRP = 26 mmHg, whereas the red solid line shows the linear regression of all measurements of patients in the IRP > 26 mmHg and IRP ≤ 26 mmHg groups. (c) IRP was higher in patients with non-dilated esophagus than in those with dilated esophagus (P < 0.01). Bars indicate median values.

Spearman's coefficient was also used to determine the correlation between IRP and age or symptom duration, revealing a statistically significant difference (-0.308 [P = 0.05] and -0.371 [p = 0.02]), respectively. IRP was higher in cases of non-dilated esophagus (P < 0.01, on Student's t-test) (Fig 5)

### Therapeutic outcomes of POEM and histology of LES

**Fig 6:**
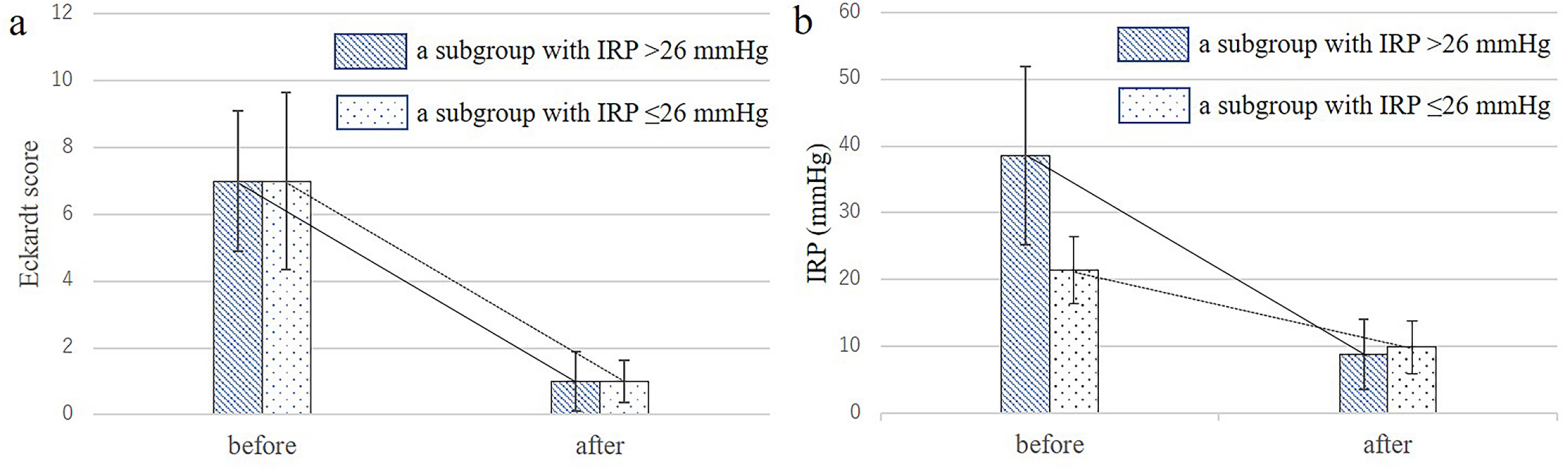
(a) Changes in the Eckardt score before and after peroral endoscopic myotomy (POEM) in the integrated relaxation pressure (IRP) > 26 mmHg group (pre-op Eckardt score 7 ± 2.1 vs. post-op 1 ± 0.9, P < 0.01) and IRP ≤ 26 mmHg group (pre-op 7 ± 2.6 to 1 ± 0.6, P < 0.01). (b) Changes in IRP (mmHg) before and after POEM in the IRP >26 mmHg subgroup (pre-operative 38.6 ± 13.3 vs. post-operative 8.9 ± 5.3 mmHg, P < 0.01) and IRP ≤ 26 mmHg subgroup (pre-operative 21.5 ± 5.0 vs. post-operative 9.9 ± 4.0 mmHg, P < 0.01).

In the IRP > 26 mmHg subgroup, a significant reduction in the Eckardt score was achieved (pre-op Eckardt score 7 ± 2.1 vs. post-op 1 ± 0.9, P < 0.01) with a significant reduction in IRP (pre-operative 38.6 ± 13.3 vs. post-operative 8.9 ± 5.3 mmHg, P < 0.01). Similarly, in the IRP ≤ 26 mmHg subgroup, the Eckardt score decreased from 7 ± 2.6 to 1 ± 0.6 (P < 0.01) with a significant decrease in IRP (pre-operative 21.5 ± 5.0 vs. post-operative 9.9 ± 4.0 mmHg, P < 0.01) (Fig 6). Between the IRP > 26 mmHg and IRP ≤ 26 mmHg subgroups, the pre- and post-operative Eckardt score and post-operative IRP were not significantly different (P = 0.36, 0.58, and 0.91, respectively).

**Fig 7:**
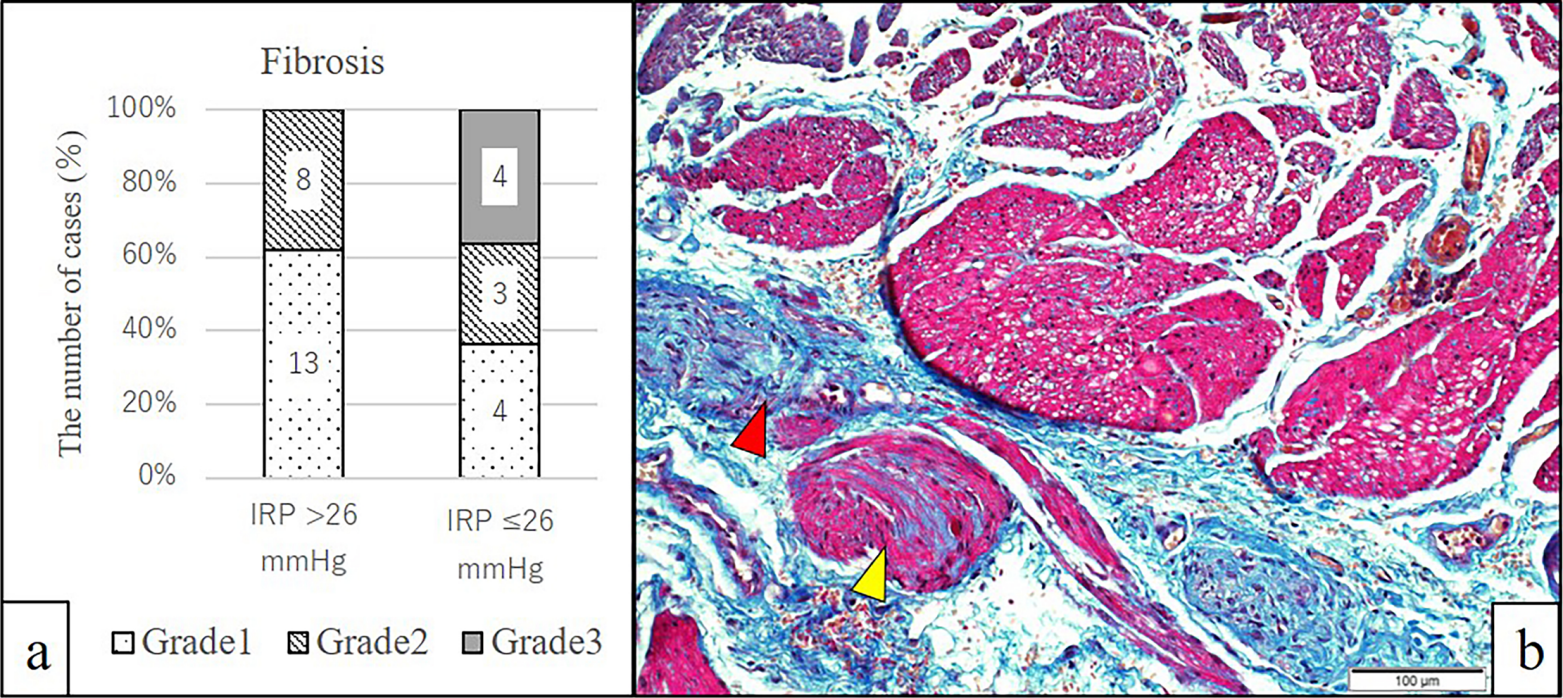
(a) Grading of lower esophageal sphincter (LES) fibrosis in the integrated relaxation pressure (IRP) > 26 mmHg subgroup (n = 21) and IRP ≤ 26 mmHg subgroups (n = 11). (b) Cases of severe fibrosis (grade 3) in the LES were only observed in the IRP ≤ 26 mmHg subgroup. Azan-Mallory staining (200×magnification) revealing severe atrophic changes with replacement by fibrosis in the smooth muscle bundles (yellow triangle). Fibrotic tissue extension in the inter-smooth muscle bundles is also seen (red triangle).

On histological assessment (histology was obtained in the latter 32 cases), severe fibrosis was only observed in four cases (12.5% of 32 cases of achalasia), which were all included in the IRP ≤ 26 mmHg subgroup (29.6% out of the 14 cases in the subgroup) (Fig 7).

## Discussion

When comparing the two subgroups of achalasia patients (IRP > 26 mmHg and IRP ≤ 26 mmHg), the same symptom severity was observed and POEM achieved significant symptom improvement in both groups. Although the range of the reduction in IRP before vs. after POEM was naturally lower in the pre-POEM IRP ≤ 26 mmHg subgroup, the post-POEM Eckardt score and IRP values converged at the same point in the subgroup with an IRP > 26 mmHg (Fig 6). Therefore, an IRP ≤ 26 mmHg can be considered as a category of achalasia characterized by an impaired LES relaxation similar to that seen in patients with an IRP > 26 mmHg. The difference between these two categories is that the IRP ≤ 26 mmHg subgroup includes more older patients and those with disease progression (longer symptom duration, more dilated esophagus and fibrosis on histology). Interestingly, even in cases with a very low IRP before POEM, POEM was effective, suggesting that impaired LES relaxation in achalasia is not only defined by HRM and that therapeutic indications should not be decided only based on HRM findings.

The use of impedance planimetry (EndoFLIP) has been recommended to assess the IRP ≤ 26 mmHg subgroup.[21] However, this expensive device is not covered by public insurance systems and is not widely used. Instead, careful endoscopic examination and esophagography were performed in this study and we confirmed that these classic modalities are a feasible substitute for impedance planimetry. When performing endoscopy, care should be taken not to induce the patient's vomiting reflex, which opens the EGJ. On esophagography, recording video allowed better evaluation of dysfunction in esophageal movement than a sequence of photographs, and timed barium esophagography [i.e. 5min] may also be useful. On HRM, EGJ relaxation is measured passively with the location of the HRM catheter fixed. Thereby, an occlusive pressure against the modality through the EGJ is necessary to assess relaxation of the EGJ,[7] which can be evaluated using endoscopy and esophagography. The major concern about using a low threshold for IRP is that it will lead to over-diagnosis of achalasia and potentially subject some patients to therapy that is too invasive, which should be avoided.

Our data demonstrated a negative correlation between IRP and aging, although the influence of aging on LES pressure is controversial based on previous studies and has still not been studied in the context of achalasia.[22, 23] The physiology of the EGJ is complex and its function depends on the interaction between several factors: LES, the crural diaphragm, sling fibers of the proximal stomach, and the phrenoesophageal ligament.[24] Aging can negatively impact some of these factors. Patients’ symptom duration and esophageal dilation were associated with LES function, and severe fibrosis in the muscle layer was only observed in the IRP ≤ 26 mmHg subgroup, suggesting that fibrosis is a result of disease progression and triggers a decline in LES pressure. Although POEM can be a feasible option even for advanced cases of achalasia, only two cases with IRP ≤ 26 mmHg, who had severe fibrosis in the esophageal muscle layer, had technical difficulties associated with POEM. Moreover, Lin *et al*. reported that the IRP cutoff value may be determined by manometry pattern and that in type-I achalasia, which has no distal esophageal contractility, the cutoff should be lower (IRP >10 mmHg for the Medtronic system).[25] The Chicago classification v3.0 does acknowledge that achalasia can be diagnosed in the absence of contractility dysfunction with IRP values < 15 mmHg. [5] In our series of achalasia patients with an IRP ≤ 26 mmHg, measured using the Starlet system, the rate of type-I achalasia was also higher compared to that in the IRP > 26 mmHg group (78.6% vs. 55.6%, Table 1), although this difference was not statistically significant. Our understanding for the observation of more type-I achalasia in the IRP ≤ 26 mmHg subgroup is that advanced achalasia changes the HRM pattern to type I from type II or III. Type-II or-III achalasia are associated with distal esophageal contractility, positively influencing LES pressure.[26]

This study has several limitations that should be discussed. First, a short-term (2 months) follow-up period was used to evaluate the outcomes of POEM. Long-term follow-up may be necessary in the future. It has been reported that the long-term outcomes of POEM are mostly good but gradually worsen over time, which may be related to disease progression preoperatively.[27] Second, for the exact evaluation of therapeutic outcomes, another parameter, instead of the Eckardt score, is necessary because it was originally proposed to assess symptom severity. Third, only one study has validated the Medtronic and starlet system in a small Japanese population. Therefore, further studies including more cases and multiple races, are needed to confirm our findings. Technical issue with the Starlet system should be also reconfirmed.

In conclusion, impaired LES relaxation in achalasia is not only defined by HRM. HRM can be used for the differential diagnosis of achalasia. In cases of suspected achalasia, comprehensive decision making is important together with endoscopy and esophagography to evaluate the occlusive pressure against the modality. Aging or disease progression are reasons for a decline in IRP in achalasia. POEM is a feasible option even for achalasia patients with normal IRP. However, some patients had severe LES fibrosis and require more attention when deciding on therapeutic options.
